# EEG Resting State Functional Connectivity in Adult Dyslexics Using Phase Lag Index and Graph Analysis

**DOI:** 10.3389/fnhum.2018.00341

**Published:** 2018-08-30

**Authors:** Gorka Fraga González, Dirk J. A. Smit, Melle J. W. van der Molen, Jurgen Tijms, Cornelis Jan Stam, Eco J. C. de Geus, Maurits W. van der Molen

**Affiliations:** ^1^Department of Psychology, University of Amsterdam, Amsterdam, Netherlands; ^2^Rudolf Berlin Center, Amsterdam, Netherlands; ^3^Department of Biological Psychology, VU University, Amsterdam, Netherlands; ^4^Neuroscience Campus Amsterdam, VU University, Amsterdam, Netherlands; ^5^Institute of Psychology, Leiden University, Leiden, Netherlands; ^6^Leiden Institute for Brain and Cognition, Leiden University, Leiden, Netherlands; ^7^IWAL Institute, Amsterdam, Netherlands; ^8^Department of Clinical Neuropsychology and MEG Center, Neuroscience Campus Amsterdam, VU University Medical Center, Amsterdam, Netherlands; ^9^Amsterdam Brain and Cognition, University of Amsterdam, Amsterdam, Netherlands

**Keywords:** electroencephalography (EEG), functional connectivity, network, graph theory, minimum spanning tree, dyslexia

## Abstract

Developmental dyslexia may involve deficits in functional connectivity across widespread brain networks that enable fluent reading. We investigated the large-scale organization of electroencephalography (EEG) functional networks at rest in 28 dyslexics and 36 typically reading adults. For each frequency band (delta, theta alpha and beta), we assessed functional connectivity strength with the phase lag index (PLI). Network topology was examined using minimum spanning tree (MST) graphs derived from the functional connectivity matrices. We found significant group differences in the alpha band (8–13 Hz). The graph analysis indicated more interconnected nodes, in dyslexics compared to typical readers. The graph metrics were significantly correlated with age in dyslexics but not in typical readers, which may indicate more heterogeneity in maturation of brain networks in dyslexics. The present findings support the involvement of alpha oscillations in higher cognition and the sensitivity of graph metrics to characterize functional networks in adult dyslexia. Finally, the current results extend our previous findings on children.

## Introduction

Reading is an intrinsically multimodal cognitive skill that requires integrated functioning of complex brain networks. We currently have a limited understanding of the neurocognitive mechanisms involved in typical reading acquisition and in the lack of reading fluency observed in dyslexic individuals (Shaywitz and Shaywitz, 2005; Dehaene et al., 2015). Several brain systems, generally localized in the left hemisphere, are found to specialize with reading expertise. These systems include auditory processing, multisensory integration and visual processing areas, their mutual interactions playing an important role at different stages of reading development (see review in Schlaggar and McCandliss, 2007). Neuroimaging studies of dyslexia have suggested deficits in activation of specific brain areas (e.g., Pugh et al., 2000a; Shaywitz et al., 2002; Froyen et al., 2011; Fraga González et al., 2014; Kronschnabel et al., 2014; Žarić et al., 2014) as well as in connectivity across the various brain systems for reading (Horwitz et al., 1998; Pugh et al., 2000b; Quaglino et al., 2008; van der Mark et al., 2011; Žarić et al., 2017). Studies using diffusion tensor imaging (DTI) suggested that dyslexics and typical readers may differ in the main white matter pathways that constitute the anatomical basis of the reading network (see review and meta-analysis in Vandermosten et al., 2012). Moreover, several studies using functional magnetic resonance imaging (fMRI) indicated that dyslexia may involve connectivity deficits between a broader range of brain networks that are not limited to those typically associated with reading (e.g., Wolf et al., 2010; Finn et al., 2014). In addition, reading abilities have been linked to abnormal resting-state connectivity between visual areas for word recognition and the dorsal attention network (Vogel et al., 2012). Finally, several studies also found differences in the organization of long-range connectivity in electroencephalography (EEG) oscillatory activity of dyslexics, suggesting reduced global efficiency (Vourkas et al., 2011; Dimitriadis et al., 2013; Fraga González et al., 2016). The present study focuses on network properties revealing the organization of widespread functional connectivity in adult dyslexics relative to the connectivity pattern exhibited by typical readers. Failure of a (reading) network can be due to abnormal organization, even when the number and strength of the connections are relatively preserved, and this scenario can be detected by network analysis.

A methodological framework to characterize complex interactions between large-scale networks is provided by the graph theory/network analysis of whole-brain resting-state data (e.g., Stam, 2014). In graph theoretical analysis, a network is represented as a set of nodes and the links (or edges) connecting them. A number of metrics can be derived from a graph to describe the topological properties of the network. These properties describe how efficiently information is communicated between the nodes and provide information on the functional “segregation” and “integration” of the networks under study (Bullmore and Sporns, 2012). Brain networks with a “small-world” topology provide an optimal balance between segregation and integration; that is, small-world topologies combine high local interconnectedness and short path length and thus provide high global efficiency (Medaglia et al., 2015; Bassett and Bullmore, 2016).

The main goal of the present study is to compare the organization of functional networks implicated in reading between dyslexics and typically reading young adults. Neuroimaging research suggests that spontaneous brain activity provides meaningful information about long-range communication between brain areas (Mantini et al., 2007; van Diessen et al., 2015). Indeed, resting-state activity has been used to map functional networks for reading (Hampson et al., 2006; Koyama et al., 2010; Vogel et al., 2012). For this purpose, we will use minimum spanning trees (MST). The MST is a special type of sub-network that was developed to facilitate such group comparisons by minimizing the bias in the computation of some network metrics (Stam et al., 2014; Tewarie et al., 2015). The MST represents a sub-network derived from a weighted network containing the highest weights possible without forming any loop or cycle. Consequently, the resulting tree always has the same number of links (*m* = *N* − 1), thus providing network comparison across groups or conditions without running the risk of bias due to differences in edge density. Importantly, the MST is proposed to represent a connectivity “backbone” capturing the main properties of the network (van Mieghem and Magdalena, 2005). Thus, the current analysis focuses on MST metrics to compare how the main network connections are organized in dyslexics compared to typical readers.

We previously used MSTs in EEG resting-state data to compare dyslexic and typically reading children in 3rd grade (Fraga González et al., 2016). In that study, we obtained significant group differences in the theta (4–8 Hz) band for two graph metrics that suggested reduced network integration and less communication between network nodes in dyslexics compared to typical readers. Two previous MEG studies compared functional networks between typical readers and children with reading difficulties (Vourkas et al., 2011; Dimitriadis et al., 2013). Vourkas et al. (2011) reported that poor readers showed reduced global and local network efficiency in the alpha band during a reading task. Dimitriadis et al. (2013), observed that reading impaired children showed reduced global efficiency in all frequency bands of resting EEG. That study also examined the temporal dynamics of sensor interdependencies and reported reduced temporal correlations in high-beta (20–29 Hz) frequencies between the temporo-parietal region and the rest of the network, i.e., reduced local efficiency, in reading impaired children. Importantly, neuroimaging research suggests that neurocognitive deficits, including connectivity abnormalities, persist in dyslexia during adulthood (e.g., Pugh et al., 2000b; Stanberry et al., 2006; Mayseless and Breznitz, 2011). Thus, the present study expands our previous line of work in children data and examines the organization of spontaneous oscillatory activity in young adults with dyslexia. Similar to our previous study, we use MST graphs aimed at detecting differences in functional connectivity organization that relate to the reading deficits.

## Materials and Methods

### Participants

Twenty-eight dyslexic young adults (23.14 ± 2.18 years old) were recruited from a nation-wide center for dyslexia in the Netherlands (see Table 1 for a summary of the main characteristics of the sample). A group of 36 typical readers (22.22 ± 2.52 years old) was recruited via the University and ads in the social networks. They had no history of reading or learning difficulties. Participants were required to have normal or corrected-to-normal vision and Dutch as their primary language. Exclusion criteria were hearing loss, diagnosis of attention deficit hyperactivity disorder (ADHD) or other neurological or cognitive impairments^1^. These criteria were ascertained by an online self-report questionnaire. This study was carried out in accordance with the recommendations of Ethics Committee of the Developmental Psychology Department, University of Amsterdam. The protocol was approved by the University of Amsterdam. All subjects gave written informed consent in accordance with the Declaration of Helsinki.

**Table 1:** Sample characteristics and descriptive statistics showing reading scores.

	Typical readers *M* (SD)	Dyslexics *M* (SD)	*F*	*p*-value	*η*^2^
*N*	36	28			
Sex ratio (m:f)	10:26	13:15			
Handedness (L:R)	1:35	3:25			
Age	22.22 (2.52)	23.14 (2.18)	2.34	0.131	0.36
RAVEN—IQ test^a^	52.19 (4.64)	51.96 (5.33)	0.03	0.854	0.00
	
One-Minute Test—*fluency*^b^	106.67 (8.96)	82.32 (14.41)	68.75	<0.001	0.52
Rapid Automatized Naming^c^					
Letters	17.04 (3.48)	21.08 (4.56)	16.15	<0.001	0.21
Numbers	18.71 (3.95)	21.14 (3.79)	6.16	0.016	0.09
Colors	25.97 (4.67)	30.90 (4.59)	17.81	<0.001	0.22
Objects	29.09 (6.00)	35.10 (5.84)	16.11	<0.001	0.21
Total	22.70 (3.41)	27.05 (3.77)	23.33	<0.001	0.27

*All raw scores. ^a^Twenty minutes time-limited version of RAVEN. ^b^Raw score = number of correctly read words within 1 min. ^c^Raw score in seconds. η^2^ = partial eta-squared*.

### Behavioral Measurements

The following tests were used to assess the reading skills and general intelligence of the participants. The tests were taken at the beginning of the session and before attaching the electrodes. Test scores are presented in Table 1.

Word reading skills were measured using a Dutch version of the *One-minute test* (*Een-Minute-Test*, EMT; Brus and Voeten, 2010) a time-limited test consisting of a list of 116 unrelated words of increasing difficulty. The number of correctly read words within 1 min serves as reading fluency score. In addition, participants completed the *rapid automatized naming* (*RAN*, van den Bos and Lutje Spelberg, 2010) task that consists of four subtasks: letters, digits, colors and objects. Per subtask a sheet is presented that contains five items repeated 10 times (arranged in a pseudo-random order). Participants are instructed to name the items as quickly as possible, and the time taken to name all items of a sheet represented the score (*r* = 0.79–0.86, split-half reliability). Finally, the *RAVEN Advanced Progressive Matrices* was used to obtain an estimate of fluid IQ (RAVEN APM; Raven et al., 1998). A 20-min timed version of this test was used as it was previously shown to be a good predictor of the untimed APM (Hamel and Schmittmann, 2006).

### EEG Measurements

#### EEG Recording and Equipment

The EEG recording took place in a dimly lit and sound-proof room. Participants were video-monitored by the lab assistants from an adjacent room to ensure they complied to the instructions and that they did not show behavioral indications of drowsiness or sleep onset during the recording. Participants were seated at approximately 80 cm distance from the computer screen. Their chair was equipped with response buttons at both arms. The EEG session started with preparation and placement of electrodes (lasting around 30 min) and continued with the baseline recording and two experimental tasks, which took around 2 h. Following the second experimental task, an additional baseline recording was performed. The current analysis is performed on the data from the baseline recordings prior to the first experimental task. During both baseline recordings subjects were required to look at the center of the screen during 4 min after making a button-press indicating the start of the baseline period. A gray background was used to minimize glare on the screen and a gray fixation circle with shadowing was placed at the center of the screen. A gray circle against a gray background was used to assist participants to fixate their eyes while preventing eye fatigue.

The EEG was recorded DC (low-pass: 5th order sync digital filter) with a 2,048 Hz sample rate. We used a 64-channel Biosemi ActiveTwo system (Biosemi, Amsterdam, Netherlands). The Biosemi system uses two additional electrodes (Common Mode Sense (CMS) and Driven Right Leg (DRL)) located to the left and right of POz, respectively, which replace the conventional ground electrode. The 64 electrodes were distributed across the scalp according to the extended 10–20 International system and applied using an elastic electrode cap (Electro-cap International Inc.). Ten external Flat-Type Active electrodes were used. Four were used to record vertical and horizontal electro-oculogram (EOG). They were placed below both eyes aligned with the pupils approximately 3 cm outside both outer canthi of the eyes. Two electrodes were placed at mastoids and two were attached to the earlobes to be used as offline reference signals. Finally, two electrodes were used to record the electrocardiogram (ECG) and were placed at the sternum and between the lower two ribs. All electrode offsets were maintained between −20 μV and +20 μV.

#### EEG Preprocessing

**Figure 1:**
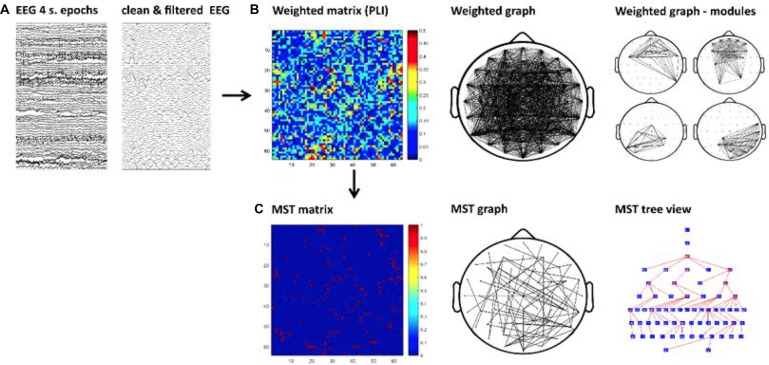
Schematic of the graph analysis. First, artifact-free epochs were Independent Component Analysis (ICA) cleaned and filtered for each frequency band **(A)**. Second, the functional connectivity matrix based on phase lag index (PLI) is calculated for each frequency band and epoch (**B**-left). A weighted graph is derived from the matrix (**B**-middle) allowing the study of modularity, which is presented in Supplementary Material (**B**-right). Finally, the Kruskal’s algorithm is applied to obtain a minimum spanning tree (MST) matrix (**C**-left). The tree can be displayed on a scalp projection (**C**-middle). The tree view shows the hierarchical structure of the graph starting from an arbitrary root node, the color map of the nodes from blue to red represents lower to higher betweenness centrality (BC; **C**-right). For illustrative purpose this figure shows the MST obtained from a single epoch in one participant.

We performed the current graph analysis following similar steps as in our previous study (Fraga González et al., 2016). A schematic of these steps is shown in Figure 1 The continuous EEG data were imported in EEGLAB v.12.5.4b, a Matlab-based open toolbox (Delorme and Makeig, 2004). The averaged earlobes were used as off-line reference when importing the data. A segment with a duration of 4 min was selected, time-locked to the button press indicating the start of the eyes-open resting-state recording. Afterwards the data were high-pass filtered at 0.5 Hz using a zero-phase FIR filter. The epoch was inspected for bad channels and those containing excessive artifacts were removed from the data and subsequently interpolated (see later in this paragraph). In the typical readers group, bad channels were removed for 16 subjects (a maximum of three channels in five subjects); in the dyslexic group, bad channels were removed in 15 subjects (a maximum of six electrodes in one subject). The data were then segmented into 60 epochs with a duration of 4 s each. We visually inspected the epochs and removed those containing artifacts such as head or muscle movements, electrode cable movement and rare jaw clinching. Subsequently, we performed an Independent Component Analysis (ICA) decomposition (Makeig et al., 1997) in order to remove blinks, eye-movements and other stereotyped artifacts from the data. The “runica” algorithm implemented in EEGLAB was used for improved detection of sources with sub-Gaussian distributions (Lee et al., 1999). We identified the independent components associated with artifacts with the automatic algorithm ADJUST (Mognon et al., 2011). The algorithm uses artifact-specific spatial and temporal features to detect artifactual components and has been previously validated (Mognon et al., 2011). The components selected by ADJUST were rejected and the data were reconstructed based on an average (SD) of 52.11 (4.80) components in the typical readers group and 51.25 (6.23) in the dyslexics group. The data from previously removed channels were interpolated using a spherical spline interpolation method (Perrin et al., 1989). Finally, a total of 30 artifact-free 4-s epochs per participant^2^ were down-sampled to 1024 Hz and exported to ASCII files.

The ASCII files were imported in Brainwave v0.9.152.4.1 (developed by C.S.; freely available at http://home.kpn.nl/stam7883/brainwave.html) where data were re-referenced to the average of all scalp channels before performing subsequent analyses. In order to explore short- and long-range connectivity across broad cortical regions, functional connectivity strength (measured with phase lag index (PLI); see Functional Connectivity) was averaged across all electrodes and across the following groups of electrodes: frontal (including the sites Fp1, Fp2, AF3, AF4, AF7, AF8, F1, F2, F3, F4, F5, F6, F7 and F8); central (FC1, FC2, FC3, FC4, FC5, FC6, C1, C2, C3, C4, C5, C6, CP1, CP2, CP3, CP4, CP5 and CP6); temporal (FT7, FT8, T7, T8, TP7 and TP8) and parietal-occipital (O1, O2, PO3, PO4, PO7, PO8, P1, P2, P3, P4, P5, P6, P7, P8, P9 and P10). These groups were further split into left and right hemisphere sites for the lateralization analysis. The same sub-averages were used to inspect regional differences in fast fourier transformation (FFT) power. It should be noted that all network metrics of MSTs were derived from the main connectivity matrix, i.e., all 64 scalp electrodes.

### Spectral Power

We calculated the spectral power using FFT with a frequency resolution of 1/4 s = 0.25 Hz. The power spectra were averaged across segments and all the groups of electrodes described in Section “EEG Preprocessing”. The power values were calculated for the following frequency bands: delta (0.5–4 Hz), theta (4–8 Hz), alpha (8–13 Hz)^3^ and beta (13–30 Hz). Relative power was computed as the ratio of the power of the corresponding band and the total power. In the current study, we excluded the gamma band (30–48 Hz) from analysis based on previous evidence suggesting that the gamma frequency range in scalp EEG recordings may be strongly affected by muscle artifact (Whitham et al., 2007), and a recent EEG report indicating low reliability of graph metrics for gamma (Kuntzelman and Miskovic, 2017).

### Functional Connectivity

The PLI was used to calculate functional connectivity between all pairs of 64 electrodes for each frequency band and segment, separately. The PLI is a measure of phase synchronization designed to reduce the effect of volume conduction by ignoring zero and π phase differences (Stam et al., 2007). The PLI measures asymmetry of the distribution of instantaneous phase differences, which are determined using the Hilbert transformation (Stam et al., 2007). A distribution that is symmetric and centered around zero may indicate spurious connectivity, and a flat distribution indicates no connectivity. Deviances from a symmetric distribution indicate dependency between sources. The PLI is obtained from time series of phase differences Δφ (*t*_k_), *k* = 1…*N* by means of:

(1) $$\text{PLI=}\left|\text{<sign[sin(Δϕ}(t_{\text{k}}))]>\right|$$

Here “sign” is the signum function. The PLI ranges between 0 and 1. A value of 0 means no coupling or coupling with a phase difference centered around 0 (mod π). A value of 1 indicates perfect phase locking at a value of Δφ different from 0 (mod π). PLI values closer to one indicate stronger nonzero phase locking.

### Minimum Spanning Trees

We calculated a MST for each PLI matrix derived per segment (see Figure 1). This approach is proposed to facilitate direct comparisons avoiding biases caused by differences in connectivity strength (e.g., Stam et al., 2014). The MST is a unique sub-graph based on a weighted matrix that connects all nodes of the network without circles or loops. The MST always contains *m* = *N* − 1 links, where *N* is the number of nodes. The MST was constructed by applying Kruskal’s algorithm (Kruskal, 1956). This algorithm iteratively selects the links with the lowest distance (i.e., lowest weights) and adds the link to the tree only if no loops are created. The result is a graph without cycles or loops in which all nodes are connected. In our MST computation, we define a link weight as 1 − PLI. Thus, the MST represents the sub-network with maximum connectivity.

There are a number of MST metrics that are used to describe the topological properties of the tree (Stam et al., 2014). We examined the following metrics, which are summarized in Table 2; degree, leaf fraction (*L*), diameter (*d*), eccentricity, betweenness centrality (*BC*), tree hierarchy (*Th*), degree correlation (*R*), kappa (*K*) and mean. The degree of a node refers to its number of links, and the *L* represents the number of nodes (*N*) on the tree with degree = 1. The leaf number has a lower bound of 2 and an upper bound of *N* − 1. The leaf number presents an upper bound to the diameter of the MST, which is the largest distance between any two nodes of the tree. The upper limit of the diameter is *d* = *m* − *L* + 2, where *m* refers to the number of links on the tree. This formula implies that the largest possible diameter will decrease with the increasing leaf number. Eccentricity of a node is defined as the longest distance between that node and any other node and is low if this node is central in the tree. The *BC* of a given node *u* is the number of shortest paths between any pair of nodes *i* and *j* that are running through* u*, divided by the total number of paths between *i* and *j*. The *BC* value ranges between 0 and 1 and relates to the importance of a node within the network. The nodes with the highest *BC* have the highest load, i.e., the highest number of shortest paths between any two nodes run through these high *BC* nodes. For example, a central node with a *BC* of one could be easily overloaded. Degree, eccentricity and *BC* are different measures for relative nodal importance and may indicate the critical nodes in a tree. The measure of tree hierarchy *T*_h_ reflects a balance between efficient communication and prevention of overload of hub nodes, reflected, respectively, by small diameter and a maximal *BC*. This balance is proposed to be important for optimal network performance (Boersma et al., 2013) and is defined as:

(2) $$T_{\text{H}}=\frac{L}{2mBC_{\max}}$$

**Table 2:** Graph metrics summary.

Graph type	Metric		Definition
Minimum Spanning Tree (MST)		Degree	Number of neighbors for a given node in the MST
	L	Leaf Fraction	Fraction of nodes with degree = 1 (leafs) in the MST
	d	Diameter	Largest distance between any two nodes of the tree
		Eccentricity	Longest distance between a reference node and any other node
	BC	Betweenness Centrality	Fraction of all shortest paths that pass through a particular node
	Th	Tree Hierarchy	A hierarchical metric that quantifies the trade-off between large scale integration in the MST and the overload of central nodes
	R	Degree Correlation	Correlation between the degrees of pairs of connected nodes
	K	Kappa	Measure of the broadness of the degree distribution (degree divergence)
		Mean	Mean weight of all edges included in the MST
Weighted graph	Lw	Path length	Path with the lowest sum of edge weights between two nodes
	Cw	Clustering coefficient	Measures the tendency to form local clusters. Likelihood that edges neighboring a node will also be connected. In weighted graphs it also accounts for the average weight of these neighbors
	Q	Modularity	Newman’s modularity index indicates how well a given partitioning of a graph can be divided into modules

*Note. The group comparison of weighted graph metrics is presented as Supplementary Material (first section)*.

The degree correlation *R* is an index of whether the degree of a node is correlated with the degree of its neighboring edges to which it is connected. The *R* is quantified by computing the Pearson correlation coefficient of the degrees of pairs of connected nodes. Kappa is the width of the degree distribution and relates to spread of information across the tree (Stam et al., 2014). High *K* indicates the presence of high-degree nodes which facilitate synchronization of the tree but also increase the network’s vulnerability if a hub is damaged (Otte et al., 2015). Finally, we computed the mean of all weights in the MST; i.e., mean connectivity in the tree.

### Statistical Analysis

We performed one-way ANOVAs for group comparisons in behavioral measures, and graph metrics. For the regional and hemispheric characterization of FFT power and PLI, we performed mixed-design ANOVAs with the within-subjects factor hemisphere (two levels; left and right hemisphere sites) and region (four levels; frontal, central, temporal, parieto-occipital), and the between-subjects factor dyslexia. In addition, since previous studies suggested that both gender (Boersma et al., 2011; Douw et al., 2011) and age (Otte et al., 2015; Smit et al., 2016) may influence network metrics, both factors were added as covariates in all group comparisons. Shapiro-Wilk tests revealed that some of the measures did not conform to a normal distribution. Control comparisons (omitted in this report for concision) were performed after a natural log transformation of the data and the pattern of significant results remained the same. Subsequently, we performed linear regression analysis between the graph metrics in which we found statistically significant group differences and behavioral measures related to reading performance and non-verbal IQ, as well as age.

## Results

### Behavioral Outcomes

The results of the ANOVAs for reading fluency and RAN are shown in Table 1. The dyslexic group performed substantially worse than typical readers on both reading, and the RAN tasks. The groups were comparable with regard to age, and the non-verbal IQ scores.

### Spectral Power

**Figure 2:**
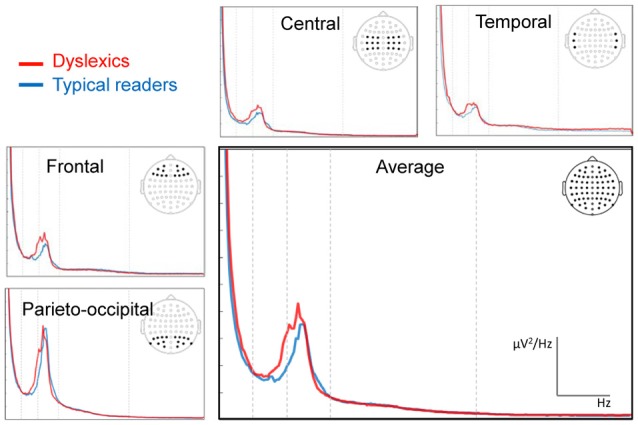
Fast fourier transformation (FFT) power spectra averaged across electrode sites for each of the sub-averages. The electrode sites for the regions frontal, central, temporal and parietal-occipital are indicated in the scalp maps. Vertical dashed lines indicate the boundaries of the main frequency bands (4, 8, 13 and 30 Hz). The red line indicates the data from dyslexic readers and the blue line those of the control group.

The spectral power plots are shown in Figure 2. We found no statistically significant (i.e., associated with a *p* value smaller than 0.05) differences between the groups in their individual alpha (range 6.50–12 Hz) peak frequencies (*p* = 0.122). The Mean (SD) peak frequencies for dyslexics and typical readers were 9.61 (1.35) and 9.13 (1.93), respectively. The analysis of relative power revealed a statistically significant effect of region in the alpha band, *F*_(3,180)_ = 4.01, *p* = 0.014, *η*^2^ = 0.07, indicating higher alpha power for parieto-occipital regions for both groups. In addition, there was a statistically significant interaction effect in alpha between the factors hemisphere, region and dyslexia, *F*_(3,180)_ = 2.74, *p* = 0.047, *η*^2^ = 0.04. Follow-up analyses revealed a statistically significant interaction between hemisphere and dyslexia for central sites, *F*_(1,60)_ = 7.45, *p* = 0.008, *η*^2^ = 0.11, indicating slightly larger alpha power in right vs. left central sites in typical readers and the reverse pattern in dyslexics. However, these hemisphere differences did not reach statistical significance in either of the groups (*p*s > 0.061). The analysis revealed no statistically significant effects of dyslexia in relative power averaged across electrodes or an interaction with hemisphere or region, for any of the frequency bands analyzed, *p*s > 0.081.

### Functional Connectivity

The analysis of connectivity strength, revealed a statistically significant main effect of dyslexia in the alpha band, *F*_(1,60)_ = 4.38, *p* = 0.041, *η*^2^ = 0.07, indicating higher PLI values in dyslexics compared to typical readers. The mean (SD) total PLI values were 0.192 (0.042) and 0.176 (0.031) in dyslexics and typical readers, respectively. There were no other statistically significant differences in PLI for any of the other frequency bands, *p*s > 0.072.

### MST Analysis

**Figure 3:**
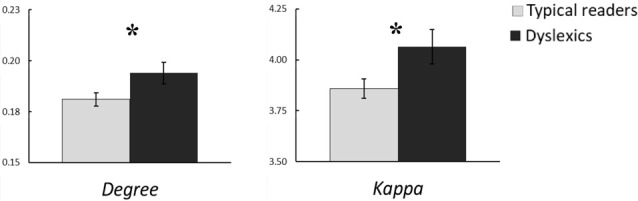
Group averages for the MST metrics degree and kappa in the alpha band. Open bars refer to typical readers and filled bars to dyslexics. *p < 0.05.

The MST analysis revealed statistically significant group differences in the alpha band (see Table 3). Compared to typical readers, dyslexics showed higher degree, *F*_(1,60)_ = 4.87, *p* = 0.031, *η*^2^ = 0.08, kappa, *F*_(1,60)_ = 5.86, *p* = 0.018, *η*^2^ = 0.09, and MST mean, *F*_(1,60)_ = 4.93, *p* = 0.030, *η*^2^ = 0.08. These differences are shown in Figure 3. In addition, there was a trend for higher BC in dyslexics relative to typical readers, *p* = 0.056. Collectively, these results suggest a more integrated tree configuration, i.e., more interconnected nodes, in dyslexics compared to typical readers. In the theta band, the group effect in MST mean approached statistical significance, *p* = 0.063. There were no other statistically significant group differences in MST metrics for any of the other frequency bands (*p*s > 0.090).

**Table 3:** MST metrics in the alpha band.

	Typical readers (*N* = 36)		Dyslexics (*N* = 28)		Group comparison		
	*M*	SD	*M*	SD	*F*	*p*-value	*η*^2^
Degree	0.181	(0.019)	0.193	(0.028)	**4.87**	**0.031**	**0.08**
Leaf	0.615	(0.024)	0.623	(0.027)	1.96	0.167	0.03
Diameter	0.206	(0.012)	0.201	(0.012)	2.92	0.092	0.05
Eccentricity	0.160	(0.009)	0.156	(0.009)	2.98	0.089	0.05
BC	0.710	(0.016)	0.718	(0.024)	*3.80*	*0.056*	*0.06*
T_H_	0.437	(0.015)	0.437	(0.015)	0.13	0.911	0.00
R	−0.354	(0.022)	−0.362	(0.024)	2.96	0.090	0.05
Kappa	3.857	(0.289)	4.061	(0.443)	**5.86**	**0.018**	**0.09**
Mean	0.454	(0.053)	0.482	(0.063)	**4.93**	**0.030**	**0.08**

*Note. Bold text represents significant results (*p* < 0.05). Italic text represents results at trend level; MST, minimum spanning tree; BC, betweenness centrality; T_H_, tree hierarchy; R, degree correlation η^2^ = partial eta-squared*.

### Relation Between Network Metrics, Age and Cognitive Performance

**Figure 4:**
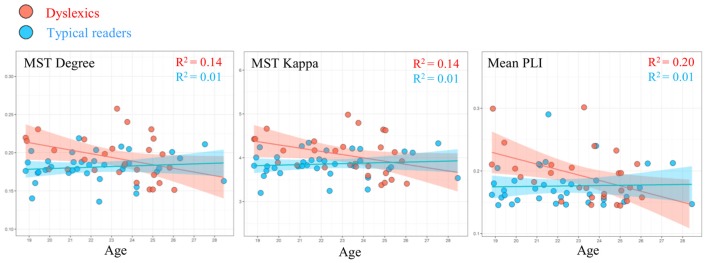
Linear regression showing the relation between age and MST metrics degree and kappa, and mean PLI in the alpha band in typical readers (blue markers) and dyslexics (red markers).

We examined the relation between the graph metrics where we found statistically significant group differences (alpha band) and age in each group separately. In the dyslexic group, the linear regression analysis showed a statistically significant relation between network metrics and age (see Figure 4). We found a statistically significant negative relation between age and MST mean (*R* = 0.46, *R*^2^ = 0.21, *β* = −0.01, *t* = −2.61, *p* = 0.015). In addition, there were trends for a negative relation between age and degree (*R* = 0.37, *R*^2^ = 0.14, *β* = −0.01, *t* = −2.204, *p* = 0.051) and kappa (*R* = 0.37, *R*^2^ = 0.13, *β* = −0.07, *t* = −2.01, *p* = 0.055). These results were absent in the typical readers group, *p*s > 0.505. We found no evidence for a statistically significant relation between graph metrics and performance on the cognitive tasks (IQ, RAN and word reading) in any of the groups, *p*s > 0.111.

Additionally, we performed the same analysis using the average PLI value and found an statistically significant relation with age in the dyslexic group, *R* = 0.45, *R*^2^ = 0.20, *β* = −0.01, *t* = −2.58, *p* = 0.016. We did not find evidence for a statistically significant relation between these variables in the typical readers group (*p* = 0.846).

## Discussion

The goal of the present study was to examine the organization of functional networks in dyslexics and typically reading adults, extending our previous findings in 3rd grade children (Fraga González et al., 2016). We used PLI to assess connectivity and MSTs to examine the topological properties of the network. Our current group comparison yielded statistically significant results mostly in the broad alpha band (8–13 Hz) that suggested group differences in strength as well as in the organization of functional connectivity.

### Network Topology Differences

The MST analysis revealed higher tree degree, kappa and mean in dyslexics compared to typical readers. This finding indicates a more integrated network configuration in dyslexics. Increased degree suggests that in dyslexics relative to typical readers, each node has on average more connections with the rest of the network (Bullmore and Sporns, 2009). The metric kappa relates to how degree is distributed within the network, i.e., degree diversity. Kappa is specially high in scale-free network models which are characterized by a few nodes (hubs) with an exceptionally high degree compared to the majority of all other nodes in the network (Stam and van Straaten, 2012). Interestingly, a previous study using MSTs on children data reported higher network integration in alpha with increased cognitive demands in a math task (Vourkas et al., 2014). That finding could imply a relation between network integration and cognitive aspects such as modulation of attentional and working memory processes. Future studies could investigate network topographies both at rest and during task to further investigate this interpretation. In addition, dyslexics showed higher MST mean, which indicates stronger connectivity, i.e., higher weights, in the tree. This finding is related to our PLI results, which are addressed later in this discussion.

The current MST results relate to potential differences in network specialization. A more integrated network configuration in dyslexics could reflect reduced presence of specialized subnetworks. The results of the weighted graph analysis presented as Supplementary Material support this interpretation as they revealed decreased modularity in dyslexics compared to typical readers. Modularity is a measure of the presence of densely interconnected nodes that form local clusters (Newman, 2006). However, caution should be exercised in interpreting the results that emerged from the weighted network analysis given its susceptibility to bias by differences in connectivity strength (see “Introduction” section). The dependence of weighted network measures on connectivity strength may be exacerbated by current group differences in PLI. Future studies may take advantage of recent developments in the analysis of MST clustering structure (Yu et al., 2015) and tree dissimilarities across the groups (Yu et al., 2016). Such analyses would contribute information about the community structure of the network while avoiding the limitations of weighted networks.

The current graph analysis revealed a more integrated network topology with stronger connections in dyslexics compared to typical readers. Our previous study reporting the results of MST analysis in dyslexic and typically reading children, we observed a somewhat different pattern of results (Fraga González et al., 2016). In that study, dyslexic children showed a less integrated tree configuration in theta, indicated by lower leaf number, degree and kappa, compared to typical readers. It is likely that age plays an important role in the different results between studies (see later discussion on the correlational results). For example, there is evidence for reorganization of connectivity in language networks due to general maturation (Brauer et al., 2011; Friederici et al., 2011) and to remediation in dyslexia (Koyama et al., 2013). In addition, theta in young children may be functionally comparable to alpha in adults; studies suggest a shift towards higher frequencies with maturation and shared topographies between higher frequencies in adults and lower frequencies in children (e.g., Niedermeyer and Lopes da Silva, 1999; Smit et al., 2012; Rodríguez-Martínez et al., 2017). This shift could explain why the results were localized in different frequency bands between this and our previous study (Fraga González et al., 2016). Besides maturation, group differences in reading practice might contribute to the apparent divergence between the current adult findings and the previous child findings. Moreover, poor readers are likely to be less exposed to print relative to typical readers (Hulme and Snowling, 2016). Future longitudinal studies should aim at disentangling maturational factors impacting EEG frequency bands.

### Group Differences in Averaged Connectivity Strength

The analysis of connectivity strength revealed higher PLI values for alpha in dyslexics compared to typical readers. In relation to this, previous EEG/MEG connectivity studies have yielded a mixed pattern of results. Some studies reported higher coherence in dyslexics (Shiota et al., 2000; Arns et al., 2007); some observed reduced coherence in poor relative to typical readers (Nagarajan et al., 1999; Dhar et al., 2010); other studies found increased coherence in some EEG bands and reduced coherence in other bands (Marosi et al., 1995). On a methodological note, coherence estimates may be strongly affected by volume conduction and reference effects, while this seems to have less impact on PLI connectivity (Stam et al., 2007). The current group differences in MST mean, reflecting the average connectivity strength of the tree, suggest that the main connections of the overall network may be implicated in dyslexia. Importantly, this result also supports the idea that MST represents a connectivity “backbone” that describes the most important network connections (Tewarie et al., 2015).

### Relation Between Network Metrics and Age in Dyslexics

We observed a statistically significant relation between several network metrics and age in the dyslexic group (see Figure 4). The most relevant weighted graph and MST metrics in the present study approached towards those of typical readers with increasing age in dyslexic participants. Age effects did not reach statistical significance levels in the typical readers group. This pattern of results suggests that compared to typical readers, the dyslexic group in current study may be more heterogeneous in their maturational trajectories of network organization (see also Fraga González et al., 2016). The direction of this relation indicates that some dyslexics individuals might show a protracted developmental course of network topology. This would be in line with the notion that dyslexic children can be characterized by a delay in the acquisition of several cognitive skills important for reading (Gallagher et al., 2000; Kuppen and Goswami, 2016). This notion is supported by neuroimaging studies of dyslexia, which suggest differences in the developmental trajectory of several neural systems specialized for reading (e.g., Hoeft et al., 2011; Maurer et al., 2011; Araújo et al., 2012; Papagiannopoulou and Lagopoulos, 2016). Furthermore, previous studies reported changes in network organization with age in children as well as in adults (Boersma et al., 2011, 2013; Wu et al., 2013; Otte et al., 2015; Smit et al., 2016). Some of these studies detected changes in MST metrics within a narrow age range in children (Boersma et al., 2013) and across a broader age range across the life span (Otte et al., 2015; Smit et al., 2016). It is possible that our current sample of young adults did not have the sufficient size and age range to detect maturational effects in network organization in both groups. Again, future longitudinal studies should further explore potential differences in the, possibly divergent, development of network topologies in dyslexics and typical readers.

### Functional Interpretation of Alpha Oscillatory Activity

The present group differences that reached statistical significance levels were confined to the broad alpha band. Oscillatory activity in alpha frequencies have been associated with cognitive and memory performance, with links to aspects such as attention and semantic memory performance (Klimesch, 1999). Another predominant line of work has related alpha to functional inhibition of task-irrelevant brain network activity (Jensen and Mazaheri, 2010). Accordingly, such task-irrelevant inhibition would facilitate the allocation of resources to task-relevant regions that are necessary for optimal task performance. Several resting-state studies reported abnormalities in the alpha band in reading-impaired children (Babiloni et al., 2012; Dimitriadis et al., 2013; Schiavone et al., 2014; Papagiannopoulou and Lagopoulos, 2016). One of those studies reported a significant correlation between a measure of global network efficiency and reading performance in typically reading children (Dimitriadis et al., 2013). Another study in adults found decreased and more diffused inter-hemisphere alpha coherence at centro-parietal sites in dyslexics relative to controls during a visuo-spatial attention task (Dhar et al., 2010). Our current findings, then, suggesting differences in the organization of alpha oscillatory activity in dyslexics further supports the relevance of these oscillations to cognitive and attentional mechanisms that may be important for optimal reading performance.

### Study Limitations

A few limitations are in order. First, the significance levels of the current results were not corrected for multiple testing. It should be noted that the graph metrics here examined are highly inter-correlated. Using spectral decomposition of the correlation matrix (Nyholt, 2004) we estimated four (instead of nine) effective variables, which would set a critical *p* value of 0.05/4 = 0.012. Although our MST effects border on medium effect sizes, their significance levels do not fall below this less stringent correction (*p*s > 0.018). Accordingly, our findings should be interpreted with caution. In the future, it is possible that this type of high dimensional data will be analyzed with machine learning tools that could be used, for instance, to perform automatic classification of subjects by group based on network features. One attractive alternative for multivariate testing with covariates is a random forest classifier (van Diessen et al., 2013). Second, there are limitations related to general aspects of the EEG montage which are common to and discussed in our previous work (Fraga González et al., 2016). A potential advantage in the current study is the use of a larger baseline recording that would arguably benefit the stability of our network metrics compared to our previous work based on a shorter recording (van Diessen et al., 2015). A related issue refers to the stability of graph measures vis-à-vis epoch length. Measures associated with relatively short epochs might be less stable, while measures obtained using longer epochs might be compromised by artifacts and non-stationarity issues. Fraschini and colleagues examined this issue and showed that PLI measures line Kappa and Ecccentricity appear to be stable using short epochs (e.g., 2 s) while other measures like Leaf and Hierarchy, reach stability using longer epochs (e.g., 6 s or longer; Fraschini et al., 2016). Here we sought a suitable compromise by using an epoch length of 4 s. Importantly, evaluating their findings, Fraschini et al. (2016) concluded that PLI-tree metrics reach good stability already at relatively short epoch lengths. Another related issue is the impact interpolation of electrodes may have on the network measures. We explored this in a control analysis in which only participants without electrodes interpolated were included (see Supplementary Material “Results” Section). The group differences between dyslexics and typical readers in MST degree and kappa remained statistically significant at *p* < 0.05, but not in PLI, MST mean and weighted network measures. Although the smaller sample size in that analysis should be considered, the results support that MST metrics provide additional information to characterize differences between dyslexics and typical readers that is not captured by connectivity strength. Moreover, the control analysis points to the impact of electrode interpolation on connectivity strength and a stronger dependency of weighted measures on PLI. Finally, there limitations regarding the anatomical interpretation and specificity of functional connectivity of EEG time series. The current methods do not allow to assess the influence of subcortical regions in the functional connectivity detected between two sensors. This could be relevant in the present case as recent studies on dyslexia suggest that deficits in subcortical hubs, such as the basal ganglia, may relate to learning difficulties experienced by dyslexics (Krishnan et al., 2016). Future graph studies may also explore the interactions between cortical and subcortical regions, possibly benefiting from source modeling techniques and MEG recordings (e.g., Boon et al., 2017).

## Conclusion

The present study extends our previous findings suggesting that global organization of functional networks in dyslexics may differ from that of typical readers (Fraga González et al., 2016). The functional connectivity results support the relevance of alpha synchronization to high-order cognition. Our graph results point to a more globally interconnected network configuration, possibly reflecting reduced functional specialization or disturbed maturation, in dyslexics relative to typical readers. This observation is in line with research suggesting that connectivity abnormalities and general oscillatory mechanisms are implicated in dyslexia. Moreover, our regression analysis suggests heterogeneity in the developmental trajectories of functional networks in dyslexia. To conclude, the current findings are consistent with emerging theoretical views and empirical evidence supporting the idea that cognitive impairments in dyslexia may result from a heterogeneous cluster of deficits rather than single local deficits (Menghini et al., 2010; Pennington et al., 2012; Pacheco et al., 2014; Fraga González et al., 2017). Future network studies of dyslexia would benefit from task-related and longitudinal data to further investigate the functional significance of these metrics and the impact of maturation on these findings.

## Author Contributions

MauritsM and EG conceived and designed the experiments. GG performed the experiments. GG, JT, MauritsM, DS and EG analyzed the data. JT, MelleM and CJS contributed reagents, materials and analysis tools. GG and MauritsM wrote the article.

## Conflict of Interest Statement

The authors declare that the research was conducted in the absence of any commercial or financial relationships that could be construed as a potential conflict of interest.

## Footnotes

^1^One subject was excluded from the initial sample of 29 dyslexics after reporting comorbidity with ADHD. From the typical readers group, two subjects from an initial sample of 38 participants were excluded due to an excessive number of segments containing artifacts in the EEG recording.

^2^Since the present analysis requires equal number of epochs across participants, we chose to include 30 epochs to avoid excluding additional subjects from analysis that may have an insufficient number of artifact-free epochs.

^3^A broad alpha range was chosen instead of the lower alpha (8–10 Hz) and upper alpha (10–13 Hz) to account for individual variability observed in alpha peak frequencies.
