# Supplementary material for: EEG Resting State Functional Connectivity in Adult Dyslexics Using Phase Lag Index and Graph Analysis
Source: Front Hum Neurosci. 2018 Aug 30;12:341. doi: 10.3389/fnhum.2018.00341 (PMC6125304; doi:10.3389/fnhum.2018.00341)
Supplement: Supplementary file 1 [file Table_1.DOCX]

Supplementary Material

EEG Resting State Functional Connectivity in Adult Dyslexics Using Phase Lag Index and Graph Analysis

**Fraga González, G. ^*^, Smit, D.J.A., Van der Molen, M.J.W., Tijms, J., Stam, C.J., de Geus, E.J.C., Van der Molen, M.W.**

*** Correspondence:** Gorka Fraga González g.fragagonzalez@uva.nl

# Weighted graph metrics

A weighted graph was obtained from the PLI matrix for each segment. In the weighted graph, each electrode represents a node and all the nodes are connected by links with a specific weight representing the strength of connectivity (Barrat, Barthélemy, Pastor-Satorras, & Vespignani, 2004). In the present analysis, we have N=64 nodes and N-1 = 63 links and the weights correspond to the PLI values. We focused on two important metrics to characterize networks clustering; that is, the clustering coefficient and the average shortest path length (see a review in Rubinov & Sporns, 2010). The clustering coefficient in unweighted networks indicates the likelihood that the neighbors of a node will be connected to each other forming a local cluster. We used the weighted equivalent of this metric, *Cw,* which also accounts for the average weight of the nodes in the local cluster (Stam et al., 2009). The average shortest path length is a measure of global integration or global efficiency. In unweighted networks, it is the number of links in the shortest path from one node to another node. We used the average weighted path length *Lw*, which represents the average highest connectivity strength of the links connecting any two nodes (this is not necessarily the path with smallest number of edges). In addition of these two metrics of local integration and global efficiency, we analyzed modularity. The modules or communities are sub-graphs within a graph, which are defined by nodes that are more strongly connected to each other than to other nodes in the network (Newman, 2006). The modularity index *Q* quantifies the community structure of the network (Newman, 2006). Modularity also relates to the notion of functional segregation or differentiation between specialized systems in the brain.

| **Table A.** Weighted graph metrics for each frequency band. | | | | | | | | | | | | |
| --- | --- | --- | --- | --- | --- | --- | --- | --- | --- | --- | --- | --- |
|  |  |  | Typical | |  | Dyslexics | |  | Group comparison | | |  |
|  |  |  | (N = 36) | |  | (N =28 ) | |  |  | | |  |
|  |  |  | *M* | *SD* |  | *M* | *SD* |  | *F* | *p-*value | *η* ^2^ |  |
|  |  |  |  |  |  |  |  |  |  |  |  |  |
| Delta | *Weighted graph* | *Lw* | 4.119 | (0.172) |  | 4.049 | (0.179) |  | 2.57 | .114 | 0.04 |  |
|  |  | *Cw* | 0.216 | (0.007) |  | 0.217 | (0.009) |  | 0.40 | .530 | 0.01 |  |
|  |  | *Q* | 0.077 | (0.002) |  | 0.077 | (0.002) |  | 0.11 | .736 | 0.00 |  |
|  |  |  |  |  |  |  |  |  |  |  |  |  |
| Theta | *Weighted graph* | *Lw* | 4.958 | (0.150) |  | 4.884 | (0.207) |  | 2.26 | .138 | 0.04 |  |
|  |  | *Cw* | 0.183 | (0.010) |  | 0.186 | (0.015) |  | 0.48 | .490 | 0.01 |  |
|  |  | *Q* | 0.078 | (0.003) |  | 0.077 | (0.004) |  | 1.82 | .183 | 0.03 |  |
|  |  |  |  |  |  |  |  |  |  |  |  |  |
| Alpha | *Weighted graph* | *Lw* | 5.031 | (0.588) |  | 4.721 | (0.646) |  | **5.21** | **.026** | **0.08** |  |
|  |  | *Cw* | 0.193 | (0.036) |  | 0.213 | (0.048) |  | **4.12** | **.047** | **0.06** |  |
|  |  | *Q* | 0.076 | (0.005) |  | 0.072 | (0.008) |  | **4.98** | **.029** | **0.08** |  |
|  |  |  |  |  |  |  |  |  |  |  |  |  |
| Beta | *Weighted graph* | *Lw* | 8.626 | (0.495) |  | 8.641 | (0.484) |  | 0.06 | .803 | 0.00 |  |
|  |  | *Cw* | 0.104 | (0.010) |  | 0.103 | (0.009) |  | 0.77 | .384 | 0.01 |  |
|  |  | *Q* | 0.079 | (0.004) |  | 0.078 | (0.005) |  | 0.44 | .511 | 0.01 |  |
|  |  |  |  |  |  |  |  |  |  |  |  |  |
| *Note.* Bold text represents significant results (*p* < 0.05).  MST, minimum spanning tree; *Lw*, weighted average path length; *Cw*, weighted clustering coefficient; *Q*, modularity; *η* ^2^ = partial eta-squared | | | | | | | | | | | | |

There was a positive relation between age and the weighted network metrics of path length (*R* = 0.50, *R^2^* = 0.25, *β* = 0.15, *t* = 2.95, *p* = 0.007) and a negative relation between age and weighted clustering coefficient (*R* = 0.46, *R^2^* = 0.21, *β* = -0.10, *t* = - 2.64, *p* = 0.014). In addition, there was a positive relation between age and modularity that just fell short of significance (*R* = 0.37, *R*^2^ = 0.14, *β* = 0.00, *t* = 2.02, *p* = 0.054).
