# Supplementary material for: EEG Resting State Functional Connectivity in Adult Dyslexics Using Phase Lag Index and Graph Analysis
Source: Front Hum Neurosci. 2018 Aug 30;12:341. doi: 10.3389/fnhum.2018.00341 (PMC6125304; doi:10.3389/fnhum.2018.00341)
Supplement: Supplementary file 2 [file Table_2.docx]

# 2 MST metrics for delta, theta and beta frequency bands

| **Table B.** MST metrics for delta, theta and beta frequency bands | | | | | | | | | | | |
| --- | --- | --- | --- | --- | --- | --- | --- | --- | --- | --- | --- |
|  |  |  | Typical | |  | Dyslexics | |  | Group comparison | | |
|  |  |  | (N = 36) | |  | (N =28 ) | |  |  | | |
|  |  |  | *M* | *SD* |  | *M* | *SD* |  | *F* | *p-*value | *η* ^2^ |
|  |  |  |  |  |  |  |  |  |  |  |  |
| Delta | *MST* | Degree | 0.164 | 0.018 |  | 0.164 | 0.020 |  | 0.20 | .653 | 0.00 |
|  |  | Leaf | 0.583 | 0.026 |  | 0.575 | 0.026 |  | 0.85 | .360 | 0.01 |
|  |  | Diameter | 0.216 | 0.009 |  | 0.220 | 0.011 |  | 1.52 | .222 | 0.03 |
|  |  | Eccentricity | 0.168 | 0.007 |  | 0.171 | 0.008 |  | 1.11 | .296 | 0.02 |
|  |  | *BC* | 0.705 | 0.015 |  | 0.706 | 0.015 |  | 0.68 | .414 | 0.01 |
|  |  | *T_H_* | 0.418 | 0.017 |  | 0.411 | 0.015 |  | 2.26 | .138 | 0.04 |
|  |  | *R* | -0.337 | 0.030 |  | -0.337 | 0.025 |  | 0.03 | .865 | 0.00 |
|  |  | Kappa | 3.562 | 0.262 |  | 3.548 | 0.285 |  | 0.05 | .817 | 0.00 |
|  |  | Mean | 0.551 | 0.025 |  | 0.561 | 0.025 |  | 2.37 | .129 | 0.04 |
|  |  |  |  |  |  |  |  |  |  |  |  |
| Theta | *MST* | Degree | 0.167 | 0.014 |  | 0.170 | 0.018 |  | 0.92 | .341 | 0.02 |
|  |  | Leaf | 0.594 | 0.022 |  | 0.594 | 0.022 |  | 0.01 | .920 | 0.00 |
|  |  | Diameter | 0.214 | 0.011 |  | 0.212 | 0.009 |  | 0.72 | .399 | 0.01 |
|  |  | Eccentricity | 0.166 | 0.008 |  | 0.164 | 0.007 |  | 1.02 | .317 | 0.02 |
|  |  | *BC* | 0.704 | 0.013 |  | 0.707 | 0.016 |  | 1.15 | .289 | 0.02 |
|  |  | *T_H_* | 0.426 | 0.016 |  | 0.423 | 0.014 |  | 0.44 | .512 | 0.01 |
|  |  | *R* | -0.344 | 0.019 |  | -0.339 | 0.019 |  | 1.28 | .263 | 0.02 |
|  |  | Kappa | 3.624 | 0.213 |  | 3.655 | 0.261 |  | 0.39 | .532 | 0.01 |
|  |  | Mean | 0.458 | 0.014 |  | 0.466 | 0.017 |  | *3.59* | *.063* | *0.06* |
|  |  |  |  |  |  |  |  |  |  |  |  |
| Beta | *MST* | Degree | 0.161 | 0.015 |  | 0.164 | 0.017 |  | 0.48 | .492 | 0.01 |
|  |  | Leaf | 0.583 | 0.024 |  | 0.584 | 0.030 |  | 0.03 | .865 | 0.00 |
|  |  | Diameter | 0.214 | 0.008 |  | 0.214 | 0.011 |  | 0.00 | .985 | 0.00 |
|  |  | Eccentricity | 0.167 | 0.006 |  | 0.166 | 0.009 |  | 0.00 | .972 | 0.00 |
|  |  | *BC* | 0.703 | 0.014 |  | 0.707 | 0.015 |  | 0.72 | .401 | 0.01 |
|  |  | *T_H_* | 0.418 | 0.017 |  | 0.416 | 0.022 |  | 0.05 | .830 | 0.00 |
|  |  | *R* | -0.324 | 0.026 |  | -0.326 | 0.037 |  | 0.12 | .730 | 0.00 |
|  |  | Kappa | 3.518 | 0.224 |  | 3.548 | 0.280 |  | 0.29 | .594 | 0.01 |
|  |  | Mean | 0.268 | 0.016 |  | 0.269 | 0.016 |  | 0.01 | .932 | 0.00 |
|  |  |  |  |  |  |  |  |  |  |  |  |
| *Note.* Italic text represents results at trend level;  MST, minimum spanning tree; *BC*, betweenness centrality; *T_H_*, tree hierarchy; R, degree correlation; *η* ^2^ = partial eta-squared | | | | | | | | | | | |
