# Supplementary material for: EEG Resting State Functional Connectivity in Adult Dyslexics Using Phase Lag Index and Graph Analysis
Source: Front Hum Neurosci. 2018 Aug 30;12:341. doi: 10.3389/fnhum.2018.00341 (PMC6125304; doi:10.3389/fnhum.2018.00341)
Supplement: Supplementary file 3 [file Table_3.docx]

# 3 Control analysis: only participants without interpolated electrodes

Participants were 13 dyslexics (23.36 ± 2.06 years old; 6 males, 7 females) and 20 typical readers (21.74 ± 2.13 years old; 5 males, 15 females). An ANOVA revealed that the age difference was significant, *F* (1, 31) = 4.71, *p* = 0.038, *η*^2^ = 0.13, indicating dyslexics were slightly older than typical readers.

Table of results:

| **Table C.** MST metrics in the alpha band for participants without electrodes interpolated. | | | | | | | | | | | |
| --- | --- | --- | --- | --- | --- | --- | --- | --- | --- | --- | --- |
|  |  |  | Typical | |  | Dyslexics | |  | Group comparison | | |
|  |  |  | (N = 20 ) | |  | (N = 13) | |  |  | | |
|  |  |  | *M* | *SD* |  | *M* | *SD* |  | *F* | *p-*value | *η* ^2^ |
|  |  |  |  |  |  |  |  |  |  |  |  |
| Alpha | *MST* | Degree | 0.179 | (0.016) |  | 0.197 | (0.033) |  | **4.98** | **.034** | **0.15** |
|  |  | Leaf | 0.616 | (0.020) |  | 0.621 | (0.029) |  | 0.69 | .413 | 0.02 |
|  |  | Diameter | 0.206 | (0.010) |  | 0.201 | (0.013) |  | 1.70 | .202 | 0.06 |
|  |  | Eccentricity | 0.160 | (0.007) |  | 0.156 | (0.010) |  | 1.97 | .171 | 0.06 |
|  |  | *BC* | 0.709 | (0.016) |  | 0.721 | (0.027) |  | 3.05 | .091 | 0.10 |
|  |  | *T_H_* | 0.438 | (0.014) |  | 0.434 | (0.010) |  | 0.52 | .477 | 0.02 |
|  |  | *R* | -0.355 | (0.020) |  | -0.362 | (0.016) |  | 0.94 | .340 | 0.03 |
|  |  | Kappa | 3.823 | (0.235) |  | 4.107 | (0.505) |  | **5.48** | **.026** | **0.16** |
|  |  | Mean | 0.457 | (0.061) |  | 0.483 | (0.070) |  | 1.72 | .200 | 0.06 |
|  |  |  |  |  |  |  |  |  |  |  |  |
|  | *Weighted graph* | *Lw* | 5.016 | (0.669) |  | 4.739 | (0.698) |  | 1.70 | .202 | 0.06 |
|  |  | *Cw* | 0.196 | (0.042) |  | 0.211 | (0.052) |  | 0.99 | .327 | 0.03 |
|  |  | *Q* | 0.076 | (0.007) |  | 0.072 | (0.009) |  | 1.59 | .217 | 0.05 |
|  |  |  |  |  |  |  |  |  |  |  |  |
| *Note.* Bold text represents significant results (*p* < 0.05)  MST, minimum spanning tree; *Lw*, weighted average path length; *Cw*, weighted clustering coefficient; *Q*, modularity; *BC*, betweenness centrality; *T_H_*, tree hierarchy; R, degree correlation;  *η* ^2^ = partial eta-squared | | | | | | | | | | | |
